# Marsupialization facilitates movement of the cystic lesion-associated deeply impacted mandibular third molar in spite of its mature roots

**DOI:** 10.4317/medoral.21814

**Published:** 2017-08-16

**Authors:** Rui Sun, Yu Cai, Yang Wu, Ji-Hong Zhao

**Affiliations:** 1 The State Key Laboratory Breeding Base of Basic Science of Stomatology (Hubei-MOST) & Key Laboratory of Oral Biomedical Engineering of Ministry of Education, School & Hospital of Stomatology, Wuhan University, Wuhan, P. R. China; 2 Department of Oral and Maxillofacial Surgery, School and Hospital of Stomatology, Wuhan University, Wuhan, P. R. China

## Abstract

**Background:**

The odontogenic cystic lesions happened in the angle and ramus region are frequently associated with impacted mandibular third molars. The treatment plan was difficult to work out for the huge cystic lesions with deeply impacted third molars, since the enucleation with simultaneously removing the deeply impacted teeth may cause serious complications. Therefore, the marsupialization of the cystic lesions followed by enucleation with tooth removal has also been advocated. The aim of this study was to explore the movement of cystic lesion-associated deeply impacted mandibular third molars (IMTM) after marsupialization.

**Material and Methods:**

Between July 2009 and December 2015, patients who had mandibular cystic lesion associated with IMTM and underwent marsupialization followed by enucleation with tooth extraction were included in our retrospective study. The clinical and pathological data was collected. The distance and direction of movement of the IMTM after marsupialization was measured on panoramic radiograph and computed tomography.

**Results:**

Four male and six female patients whose ages ranged from 14 years to 67 years were enrolled in this study. Among the all impacted molars, there were 3 cases with mature roots. After marsupialization, all the cystic lesions shrunk and all impacted teeth moved toward the bony windows, and the distance of tooth movement were from 8.3mm to 12.1mm. The complications included swelling and pain, while no numbness of the ipsilateral lower lip was happened.

**Conclusions:**

Marsupialization can promote the movement of impacted teeth with or without mature roots, and may be an optimal treatment approach for the huge posterior mandibular cystic lesions with deeply impacted third molar.

** Key words:**Mandibular cystic lesion, impacted third mandibular molar, marsupialization, mature root.

## Introduction

The variant types of odontogenic cystic lesions, including dentigerous cyst (DC), keratocystic odontogenic tumor (KCOT) and unicystic ameloblastoma (UA), frequently encountered the posterior mandible region, typically in the angle and ramus region (1). According to the WHO histological classification of odontogenic tumours, about one-half of KCOTs originate at the angle of the mandible and more than 90% of UAs involve the mandibular posterior region. These cystic lesions are often associated with impacted mandibular third molars, some reports indicated that up to 80% of these lesions are associated with an unerupted mandibular third molar (2,3). The standard treatment for those lesions is enucleation and extraction of the involved teeth, but the treatment plan was difficult to work out for the huge cystic lesions with deeply impacted teeth. In these circumstances, the enucleation with simultaneously removing the impacted teeth may cause the nerve injury or pathological mandibular fracture during the surgery. Therefore, the marsupialization of the cystic lesions followed by enucleation with tooth removal has also been advocated by some clinicians, since it can decrease the lesion size, promote new bone formation and induce the eruption of the impacted tooth (4,5).

However, the limited successful cases have been reported in the literatures, and Sano et al. (4) also indicated that this two-stage surgical procedure could not facilitate the eruption of the tooth with mature roots. Therefore, to investigate the effect and complications of the marsupialization followed by enucleation with tooth removal, we retrospectively review the cases of cystic lesions in the posterior mandible treated with this surgical procedure in our departmentydepartment.

## Material and Methods

- Inclusion criteria

**Figure 1:**
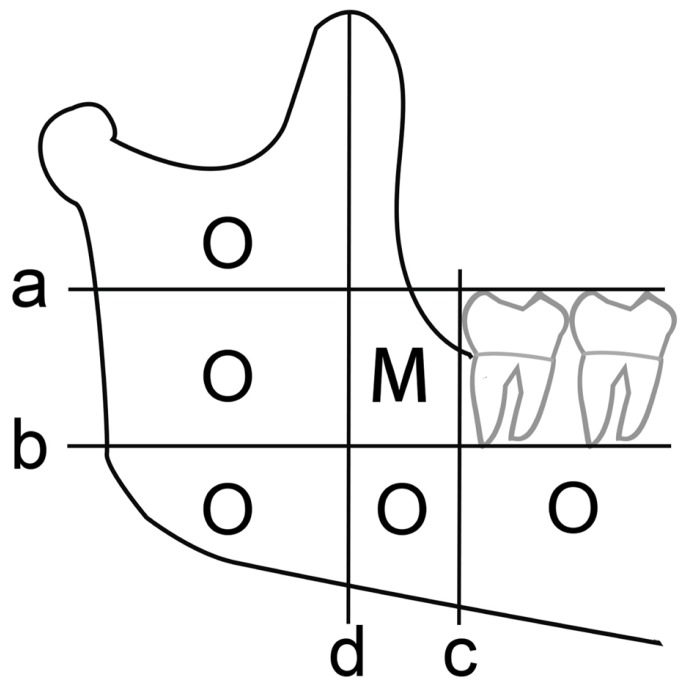
The ramus and posterior mandible would be divided into several regions by some lines. a: Occlusal plane of the mandibular 2nd molar; b: The line parallel to (a), through the apex of intact roots of the nearest mandibular molar; c: Tangent line of the distal contour of the second molar and perpendicular to (a); d: The line located distal to and parallel to (c), while the distance between (c) and (d) is equal to the length of the mesio-distal width of the 1st molar. We presume the region marked by “O” is the normal position of mandibular 3rd molar, and the regions marked by “X” are help to assess whether an IMTM can be classified as “deeply impacted tooth”.

Patients were enrolled into our study as long as they were diagnosed as having cystic lesion-associated deeply IMTM through the clinical, radiographic, and pathologic findings. The ramus and posterior mandible were subdivided into several regions in order to assess the position of IMTM on panoramic radiograph, and we defined “deeply IMTM” as the whole tooth was displaced far away from its normal position which had been marked by “XO” (Fig. 1).

- Exclusion criteria

1. Loss of ipsilateral mandibular first or second molar.

2. All the roots of ipsilateral mandibular molars were absorbed which could reduce the accuracy of dividing regions.

3. Cystic lesions accompanied by mandibular fracture were also expelled.

- Approach and technique

Marsupialization was performed under local anesthesia, and the second molar was removed if the root absorption had happened. A bony window was made by enlarging the tooth socket if the second molar had been removed or on the distal alveolar ridge of the second molar if the second molar had not been removed. During the marsupialization procedure, a biopsy was performed to confirm the histopathologic diagnosis. Finally, iodoform gauze was packed into the cystic cavity in order to hemostasis and antiphlogosis.

Ten days after surgery, the gauze was removed and a customized acrylic stent was made as space maintainer to keep the fenestration open. All patients were asked to use the stent insistently and return visit every 3 months. Panoramic radiography or CBCT was performed every attendance to investigate the bone formation and the location of the IMTM.

- Measurements

**Figure 2:**
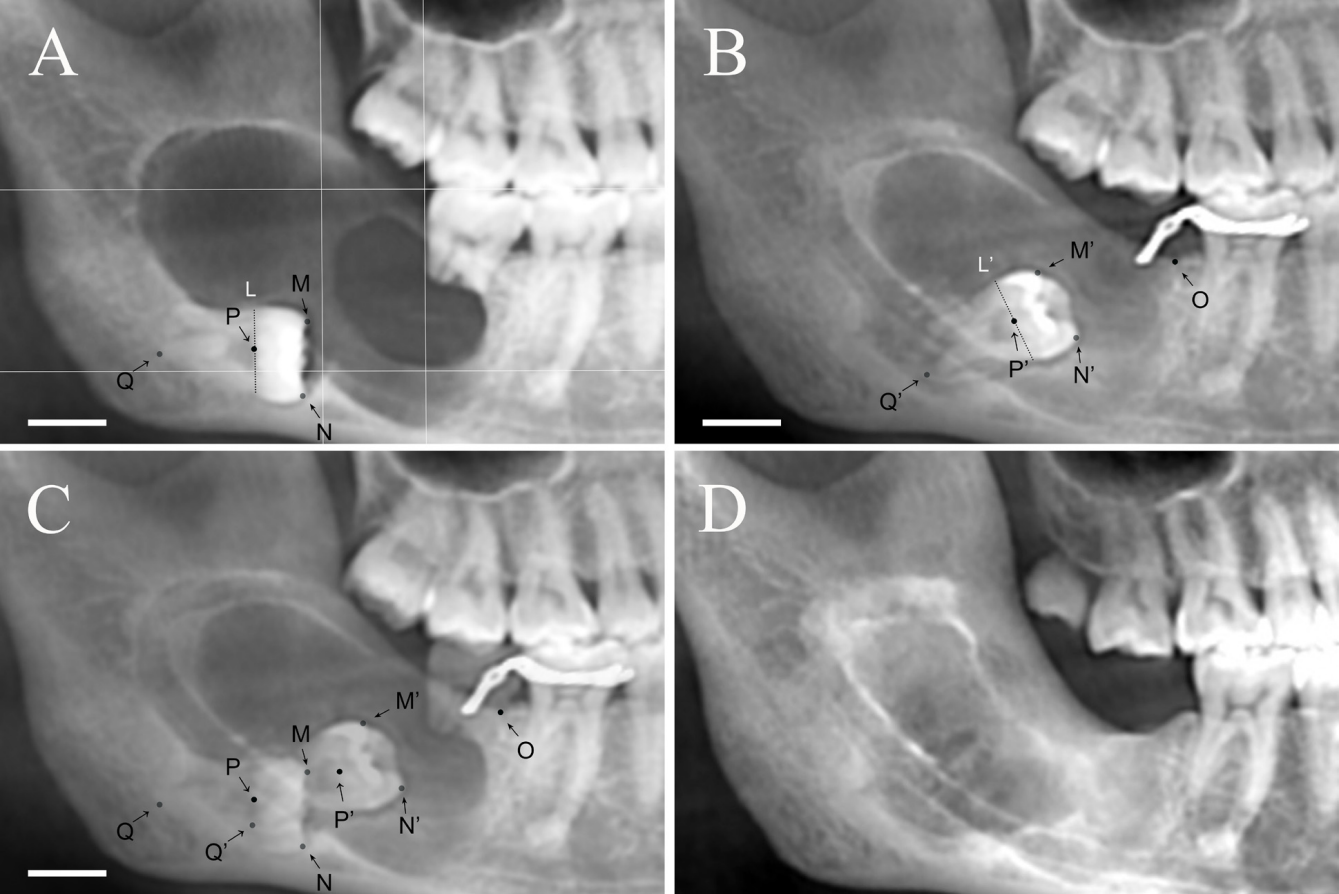
(A-C) Measurement of movement on panoramic radiograph. A: Initial panoramic radiograph, dental cusps of IMTM are marked as point (M) and (N), midpoint of the tooth is marked as point (P) which is also the midpoint of phantom line (L), line (L) divide the tooth into anatomical crown and anatomical root, root tip is marked as point (Q); B: Last panoramic radiograph, the position of the IMTM has already changed, point (M’) , (N’), (P’), (Q’) and line(L’) correspond to point (M) , (N), (P), (Q) and line(L) successively, the mesio-point of bony window is marked as point (O); C: Merged image for measurement. All scale bars: 10mm. (D) CBCT showed no recurrence 2 years after the enucleation with tooth removal.

The age and gender of the patients and the root formation of the IMTM were recorded. The distance and direction of movement of IMTM was measured. We merged the initial panoramic radiograph and the last panoramic radiograph into one. Some points which could be recognized in both initial and last radiograph were highlight in order to label cusps, midpoints, and root tips. Bony windows were regarded as reference points (Fig. 2A-C).

The length of M-M’, N-N’, P-P’, Q-Q’, O-M, O-M’, O-N, O-N’, O-P, O-P’, O-Q, O-Q’ were measured. The measurement was repeated 4 times, with longer than a 3-days interval between each measurement. The agreement of the two investigators with regard to the measurement of length was assessed using Cohen’s kappa test.

**Figure 3:**
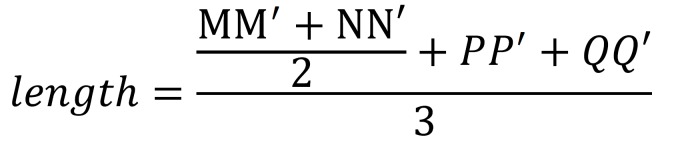
.Formula.

The calculation formula of the length of movement is illustrated as follow: (Fig. 3).

**Figure 4:**
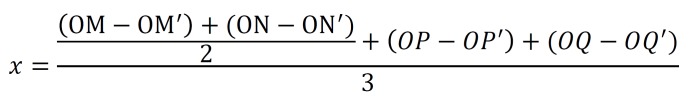
Formula.

Another formula for analysist the relative movement which regarded bony window as reference is as follow. We considered the ITMT moved toward to the bony window if x>0. Otherwise, the ITMT didn’t move toward to the bony window, (Fig. 4).

**Figure 5:**
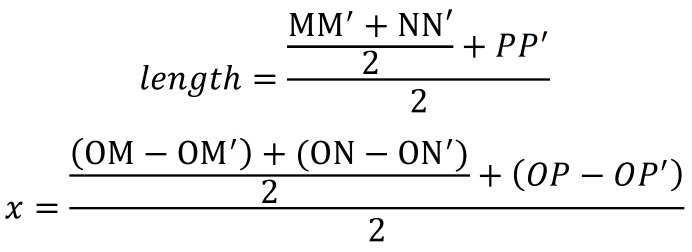
Formula.

If the crown has formed completely while the roots still in the stage of formation. The points label the root tips would be neglected. The formulas would be applied as follow: (Fig. 5).

**Figure 6:**
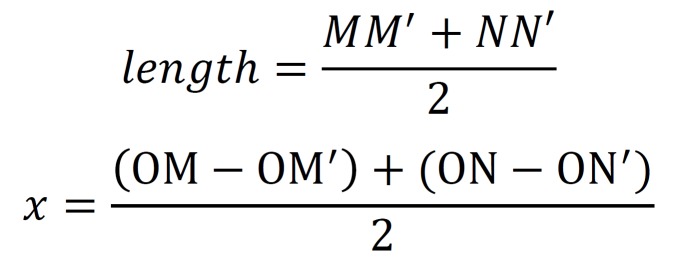
Formula.

If the crown has not formed entirely, the points label the midpoints or the root tips would be neglected. The formulas would be used as follow: (Fig. 6).

The study was approved by the review board of the Ethics Committee of the Hospital of Stomatology, Wuhan University. Because of the retrospective nature of the present study, the Ethics Committee of the Hospital of Stomatology of Wuhan University granted an exemption in writing for informed consent.

- Secondary adjunctive treatment

According to the size of the lesion, the location of the IMTM and the willingness of the patient, the secondary adjunctive treatment would be performed and recorded. The resected tissue was routinely processed for histologic assessment. Information concerning the response to treatment and any complications after surgery was obtained from case notes and follow-up data.

## Results

Between July 2009 and December 2015, a total of 10 patients with the mandibular cystic lesion with deeply IMTM were prospectively enrolled for the treatment with marsupialization followed by enucleation with tooth removal at the Department of Oral and Maxillofacial Surgery, Hospital of Stomatology, Wuhan University (the clinical data see  Table 1). There were 4 males and 6 females in this series. The ages ranged from 14 years to 67 years, and the mean age was 22.8 years. The primary sites were present in the left mandible in 6 cases, while 4 in the right.

**Table 1:**
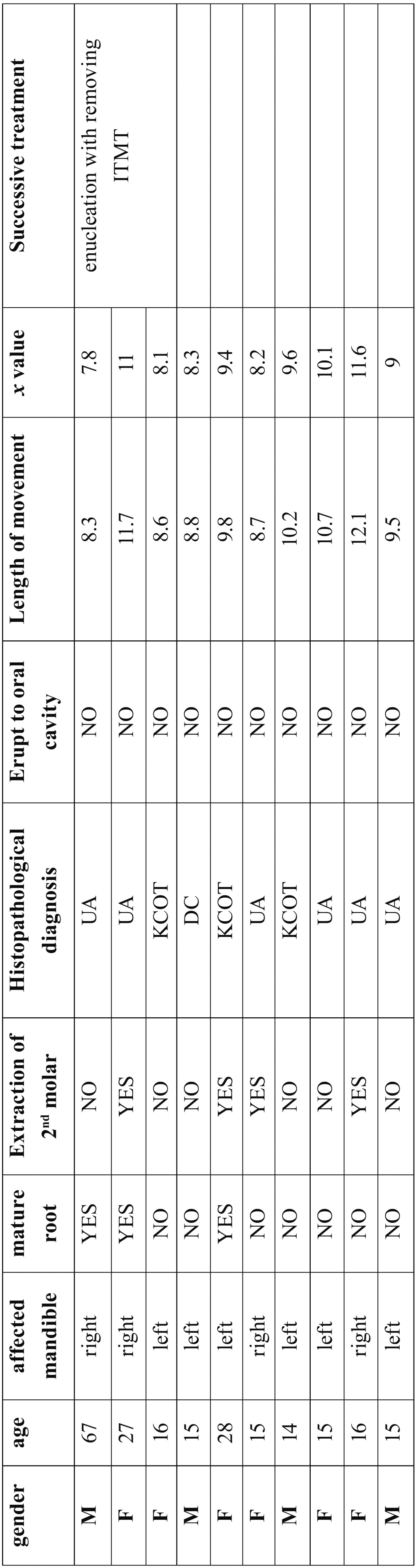
The clinical, radiographic and histopathologic data of the patients.

Among the 10 patients, 6 cases were histopathologically diagnosed as UA, 3 cases were KCOT and 1 case was DC. All cystic lesions were gradually decreased and all impacted third molars including the three with mature root ( Table 1 and Fig. 2) moved toward the bone windows after marsupialization. The mean length of movement of IMTM was 9.84mm.

In this series, the intervals between the marsupialization and enucleation were from 10 to 18 months, and most of the patients accepted the secondary surgery about 12 months after the marsupialization. The patient who was 67-year-old was received the 2nd surgery 18 months later. Median duration of follow-up was 2 years (range, 13 months to 29 months). Overall, no recurrence had been found till their last visit (Fig. 2D).

Slight or moderate swelling and pain occurred in the location of fenestration, and these symptoms resolved 5 to 7 days. After the secondary surgery, no numbness of the ipsilateral lower lip was occurred. Furthermore, the bone fracture of mandible was never happenhappened. There were no other complications associated with this surgery modality.

## Discussion

Marsupialization is an effective superior approach and has been widely applied for dental cysts as well as cystic benign bony tumors (6). It can decrease the cystic lesion size, reduce the incidence rate of recurrence and injure to important anatomical tissue nearby and bone tissue, facilitate new bone formation and the involved teeth to erupt (5). In present study, the size of the all cystic lesions was obviously shrunk and the new bone was formed after the marsupialization. And there was another exciting result that all the IMTMs with or without mature roots moved toward the bony windows, which facilitate the teeth extraction during the second stage surgical treatment.

Tooth eruption is both a physiological and developmental phenomenon of a tooth from the site of development to its functional position in the oral cavity (7). Unlike normal situation, the cystic lesion associated teeth could not erupt normally as a result of the additional resistance to the involved teeth from the lesion. In 1993, Kokich *et al.* (8) indicated that an impacted tooth without complete root formation or with an open apex had considerable potential to erupt after marsupialization. Hereafter, many cases reported marsupialization had facilitated eruption of cystic lesion-associated impacted tooth. However, no case was relevant to the tooth with complete root formation. Some researchers (4,9,10) believed that if the dental root had been mature, the tooth might not erupt to the normal position after marsupialization. In present study, we verdict that teeth with mature roots have motive force to eruption in spite of they might not have the ability to move to the normal position.

About the motive force to normal tooth eruption, Wise *et al.* (7) summarized four origin: alveolar bone formation, root elongation, periodontal ligament and vascular pressure. The IMTM with open apex may also have these four motivations. As to the 3 cases with mature roots, the formation of new bone and the reduction of intracystic pressure after marsupialization could promote the eruption of the impacted teeth.

However, all the impacted teeth in this series were unable to erupt to the normal position. The modality of bone formation may be one reason: The new bone formation, which was from the periphery of lesion to center after marsupialization, played complicated roles in different stage of the movement of cystic lesion associated-IMTM. In the initial stage, new bone formation promoted the movement of IMTM while no major resistance was present; as time went on, the expanding contact area between bone and tooth slowed down the movement; lastly, the new formation bone may prevent the movement of the tooth. Additionally, in some cases the cystic plug also may be a reason which influenced the movement of the teeth.

Taken together, marsupialization followed by enucleation with tooth removal is an optimal treatment approach for the mandibular cystic lesion with deeply impacted third molar, whether the roots are mature or not. Furthermore, if the impacted teeth need to be preserved in some circumstance, the orthodontic treatment should be considered to tract the IMTM to the suitable position.
